# The clinical efficacy of auricular acupuncture in the treatment of frequent episodic tension-type headache: a single-blind randomized controlled trial

**DOI:** 10.1186/s12906-025-05063-x

**Published:** 2025-08-21

**Authors:** Xiaxia Jin, Ziwen Xu, Tao Gao, Gaofeng Wang, Wendi Dong, Junyi Jin, Yongmei Yan

**Affiliations:** 1 National Resource Center for Chinese Materia Medica, China Academy of Chinese Medical Sciences, Beijing, 100700 China; 2 Swiss University of Traditional Chinese Medicine, Bad Zurzach, 5330 Switzerland; 3 Shaanxi University of Chinese Medicine, Xianyang, 712046 China; 4 Affiliated Guangdong Hospital of Integrated Traditional Chinese and Western Medicine of Guangzhou University of Chinese Medicine, Foshan, Guangdong 528200 China; 5 Lishui People’s Hospital, Lishui, 323000 China; 6 Affiliated Hospital of Shaanxi University of Chinese Medicine, Xianyang, 712000 China

**Keywords:** Auricular acupoints, Frequent episodic tension-type headache, Auricular acupuncture, Clinical efficacy

## Abstract

**Background:**

Frequent episodic tension-type headache (FETTH) is a significant public-health concern. Scientific research has demonstrated that acupuncture can improve the clinical symptoms of FETTH.

**Purpose:**

This study aimed to compare the efficacy and safety of auricular acupuncture and sham acupuncture in treating FETTH.

**Methods:**

This was a randomized controlled trial. Participants with FETTH were randomly assigned in a 1:1 ratio to receive auricular acupuncture or sham acupuncture for 4 weeks, with follow-up lasting for up to 24 weeks. The primary outcomes were measured using the headache index and visual analog scale (VAS) score. Secondary outcomes included the Hamilton Anxiety Scale (HAMA) score, Hamilton Depression Scale (HAMD) score, blood flow velocity in the anterior cerebral artery (ACA), middle cerebral artery (MCA), and posterior cerebral artery (PCA) (V_ACA_, V_MCA_, and V_PCA_, respectively), and the usage of acute headache medications. Adverse events were also recorded to assess safety.

**Results:**

Compared with the sham acupuncture group, the auricular acupuncture group showed significant improvements in VAS score (2 (1.25, 2) vs. 3 (2, 3.5) at 24 h, *P* < 0.001; 2 (1.25, 2) vs. 2 (2, 3) at the 2nd week, *P* = 0.004; 2 (1, 2) vs. 2 (1.5, 3) at the 4th week, *P* = 0.015), headache index (4 (2, 7) vs. 7 (4, 9) at the 4th week, *P* = 0.016), HAMA score (11.69 ± 2.70) vs. (14.24 ± 3.20) at the 4th week, *P* < 0.001; (9.83 ± 2.71) vs. (11.95 ± 2.59) at the 8th week, *P* = 0.001; (9.67 ± 2.65) vs. (11.76 ± 3.00) at the 24th week, *P* = 0.002), HAMD score ((14.25 ± 2.68) vs. (15.89 ± 3.48) at the 4th week, *P* = 0.027,) V_ACA_ (85.5 (85, 86) vs. 83 (83, 84) at the 4th week, *P* < 0.001), V_PCA_ (82 (81, 83) vs. 78 (77, 79) at the 4th week, *P* < 0.001), and usage of acute headache medications (8 (7, 10) vs. 9 (8, 11) at the 4th week, *P* = 0.030). The incidence of adverse events was similar between the two groups (*P* = 1.000).

**Conclusion:**

This study found that auricular acupuncture effectively improved the clinical symptoms of FETTH and had relatively fewer side effects. Trial registration: The study was retrospectively registered in the International Traditional Medicine Clinical Trial Registry (ITMCTR2025000363) on January 20, 2025.

## Introduction

Tension-type headache (TTH) is a recurrent primary headache disorder characterized by bilateral pain of mild to moderate intensity that typically lasts from 30 min to 7 days [1]. TTH is the most common type of primary headache and one of the most prevalent neurological disorders in the general population [2]. A global survey on the prevalence of headaches found that the overall prevalence of headaches was approximately 52.0%, with TTH accounting for about 26.0% of these headaches [3]. The annual prevalence of Frequent Episodic Tension-Type Headache (FETTH) is approximately 21.6% [4]. FETTH is characterized by frequent episodes of headache that are typically bilateral, with a pressing or tightening quality and mild to moderate intensity. These headaches may last from minutes to days. The pain does not worsen with routine physical activity and is not associated with nausea. However, photophobia or phonophobia may be present. FETTH is diagnosed by at least 10 episodes of headache occurring on 1 to 14 days per month, repeated over a period of at least three months (≥ 12 days and < 180 days per year) [5]. Pericranial myofascial nociception may play a significant role in the pathophysiology of FETTH, whereas sensitization of central nociceptive pathways appears to be responsible for the transition from episodic to chronic TTH [6]. Furthermore, 60% of TTH patients report that the pain significantly impacts their daily life, work, and social activities. Therefore, early and timely intervention is particularly important [7].

Non-steroidal anti-inflammatory drugs (NSAIDs) and antidepressants are the main Western pharmacological treatments for TTH. However, these medications often cause adverse drug reactions and poor patient compliance. Specifically, side effects such as medication-induced headache exacerbation, medication-overuse headache, and drug dependence due to long-term high-dose use are of particular concern. Traditional Chinese medicine (TCM) provides alternative approaches for TTH. Acupuncture, a non-pharmacological therapy, has demonstrated efficacy in treating TTH [8]. Among these approaches, auricular acupuncture stands out for its high safety, low cost, and minimal adverse reactions [9]. In this study, we aimed to provide solid evidence to demonstrate the efficacy of auricular acupuncture in the treatment of FETTH.

## Methods

### Study design

This study was designed as a randomized controlled trial (RCT), which is widely recognized as a reliable research method in clinical research. A total of 80 participants were included and then randomly divided into the treatment group and the control group, with 40 participants in each group. The treatment group received auricular acupuncture therapy, while the control group received sham acupuncture therapy. This design ensured that the two groups were identical at the beginning of the study, thereby minimizing the effects of selection bias and confounding factors.

The trial was designed and reported in accordance with the Standards for Reporting Interventions in Clinical Trials of Acupuncture (STRICTA) and the Consolidated Standards of Reporting Trials (CONSORT) guidelines, both of which are crucial for ensuring the transparency and reproducibility of the research. This project was approved by the Ethics Committee of the Affiliated Hospital of Shaanxi University of Chinese Medicine (Ethics Approval No. SZFYIEC-PJ-2023-27). The study was planned in accordance with the Declaration of Helsinki. All participants gave written informed consent before participating in this study.

### Participants

**Fig. 1:**
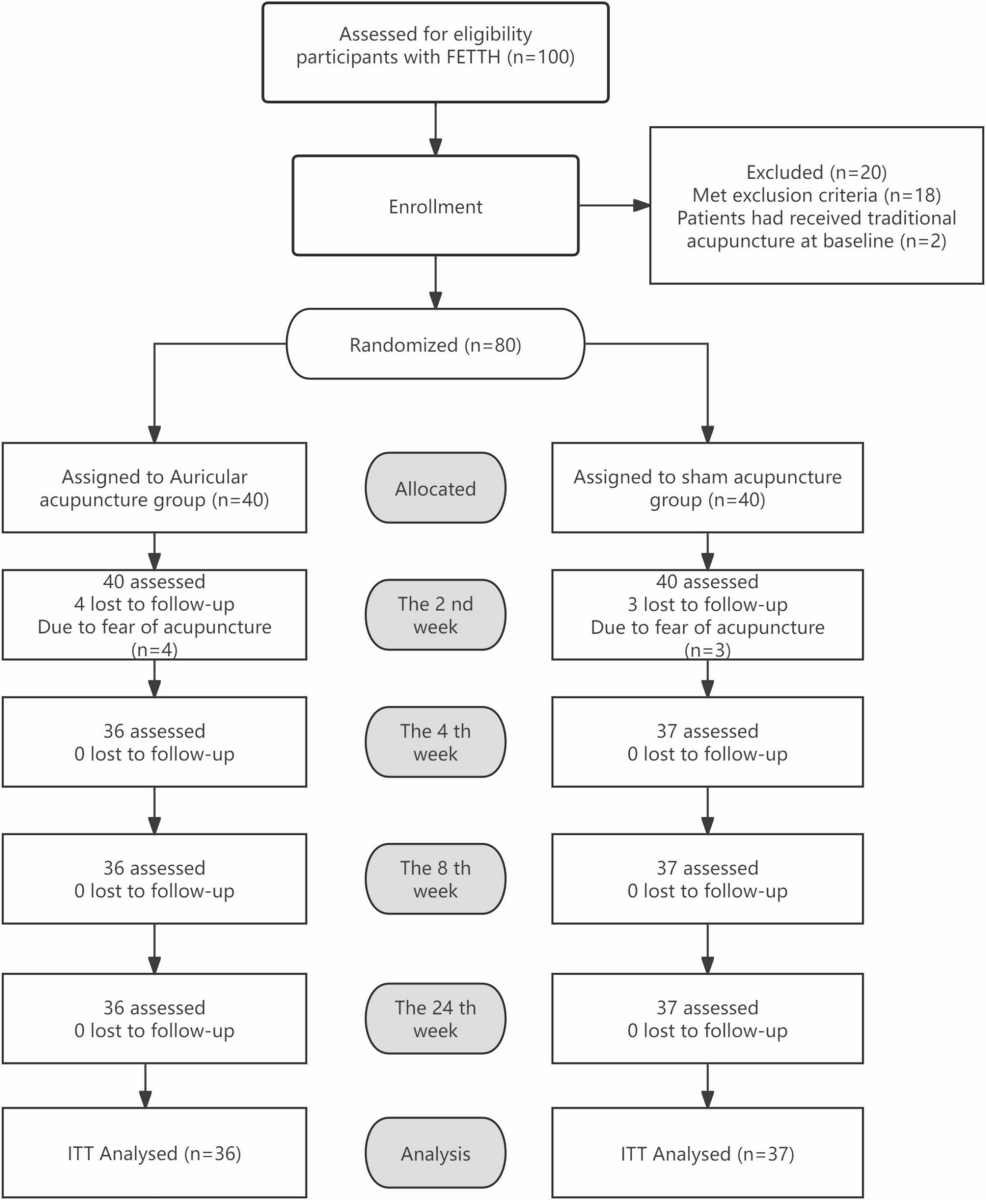
Clinical trial flow chart: Participant Flow Diagram. A total of 100 participants underwent initial screening for eligibility. Of these, 20 were excluded based on predefined criteria. The remaining 80 eligible participants were randomly assigned to either the treatment group (*n* = 40), which received auricular acupuncture, or the control group (*n* = 40), which received sham acupuncture. Four participants in the treatment group and three in the control group discontinued the intervention or were lost to follow-up. The final analysis included 73 participants, with efficacy and safety assessments adhering to the intention-to-treat (ITT) principle

Participants were recruited from outpatients of the Department of Acupuncture and Tuina and the Department of Encephalopathy at the Affiliated Hospital of Shaanxi University of Chinese Medicine between May 2023 and April 2024. The clinical trial flow chart is shown in Fig. 1.

### Sample size estimation

A randomized controlled trial design was used in this study. The sample size was calculated using the following formula:

$$\color{black} {\mathrm n\;=\;\frac{2\left({\mathrm z}_{\mathrm a}+{\mathrm z}_\beta\right)^2\;\ast\mathrm\sigma^2}{\mathrm\delta^2}}$$

At an α level of 0.05, we obtained Z scores of Z_*α*_ = 1.96 and Z_*β*_ = 0.84 from the standard normal distribution table. Based on previous RCTs on auricular acupuncture treatment for headache, we hypothesized that the mean Visual Analog Scale (VAS) score in the auricular acupuncture group would be 3 points lower than that in the sham-acupuncture group (mean difference, δ = 3) [10]. The standard deviation (σ) was set at 4.5, reflecting the variability in pain intensity among participants as reported in previous studies [10, 11]. Considering a 20% attrition rate, we calculated that we needed to recruit at least 88 participants to ensure the study’s power and validity.

### Blinding of methods

Randomization was performed by the designated investigator using a random-number generator in SPSS 25.0. After eligible participants were identified based on the selection criteria, the recruiter obtained the randomization number from the designated investigator and assigned participants to the treatment or control groups. To prevent communication between the two groups, team members responsible for each group were assigned to separate wards.

Registered traditional Chinese medicine physicians with at least 3 years of clinical experience were involved in the study and trained to treat participants according to the study protocols. Physicians were aware of each participant’s group assignment, whereas participants and assessors were unaware of the group assignments. At the end of the treatment, participants were asked about their perceived treatment allocation to evaluate the success of the blinding procedure.

### Treatment credibility

To assess the reliability of treatment blinding and the credibility of the assigned treatment groups, a credibility questionnaire was administered at each follow-up evaluation. This questionnaire was designed to explore participants’ perceptions of whether they had received auricular acupuncture or sham acupuncture. After the study was completed, participants were also surveyed about their rationale for the treatment and their willingness to recommend the assigned intervention. The Kaiser-Meyer-Olkin (KMO) measure of sampling adequacy and Bartlett’s test of sphericity were used to validate the questionnaire. The KMO value exceeded 0.6, and Bartlett’s test was statistically significant (*P* < 0.05), confirming the questionnaire’s suitability for factor analysis. The James Blinding Index (JBI) and Bang Blinding Index (BBI) methods were used to evaluate the implementation of the blinding procedure and quantify the reliability of the test results.

### Statistical analysis

Statistical analysis was conducted using SPSS Statistics 26.0. A difference was considered statistically significant if the two-tailed *P* value was less than 0.05. Efficacy and safety were analyzed based on the intention-to-treat (ITT) principle. Baseline characteristics were expressed as mean (standard deviation, SD). For continuous variables, t-tests were used for normally distributed data, while the Mann-Whitney U test or Kruskal-Wallis test was used for non-normally distributed data to analyze baseline differences between groups. For categorical variables, chi-square tests or Fisher’s exact tests were conducted.

### Diagnostic criteria

The FETTH diagnostic criteria according to the 2018 International Classification of Headache Disorders (ICHD-3) [12] are as follows: (1) Frequency and Duration: Average headache duration of ≥ 1 day/month but < 15 days/month over a period of ≥ 3 months (≥ 12 days/year but < 180 days/year). OR There are ≥ 10 headache episodes fulfilling diagnostic criteria (2)–(4) below. (2) Each headache episode lasts ≥ 30 min and ≤ 7 days. (3) Headache has at least two of the following characteristics: ① Bilateral location; ② Characterized by a pressing or tightening quality (non-pulsating); ③ Mild or moderate intensity; ④ Not aggravated by routine physical activity (e.g., walking or climbing stairs). (4) Both of the following are present: ① No nausea or vomiting (appetite may be diminished); ② Photophobia or phonophobia may be present, but not both; (5) The headache cannot be better explained by another ICHD-3 diagnosis.

### Study inclusion and exclusion criteria

#### Inclusion criteria

Participants were included in the trial if they met the following criteria: (1) They met the aforementioned FETTH diagnostic criteria; (2) They were aged from 18 to 65 years; (3) They agreed to receive acupuncture treatment for at least 4 weeks; (4) They signed informed consent to participate in this clinical trial and agreed to be followed up after treatment.

#### Exclusion criteria

Participants were excluded from the trial if they met the following criteria: (1) They had allergies, particularly to metals or adhesive tape; (2) They were pregnant or breastfeeding; (3) They had mental or cognitive impairments; (4) They had primary organic diseases (e.g., cardiovascular, hepatic, pulmonary, renal, or other major organ diseases); (5) They had secondary headaches or other types of primary headaches (e.g., migraine, cluster headache).

#### Dropout criteria

Participants were withdrawn from the trial if they met the following criteria: (1) If a serious cerebrovascular condition occurred, the participant was withdrawn from the trial and advised to seek appropriate treatment after physician evaluation; (2) If complications occurred after receiving acupuncture treatment, the participant was withdrawn from the trial and recommended to seek appropriate treatment after physician evaluation; (3) If participants could not continue for personal reasons, they were withdrawn from the trial.

### Interventions

#### The acupuncture prescription

**Fig. 2:**
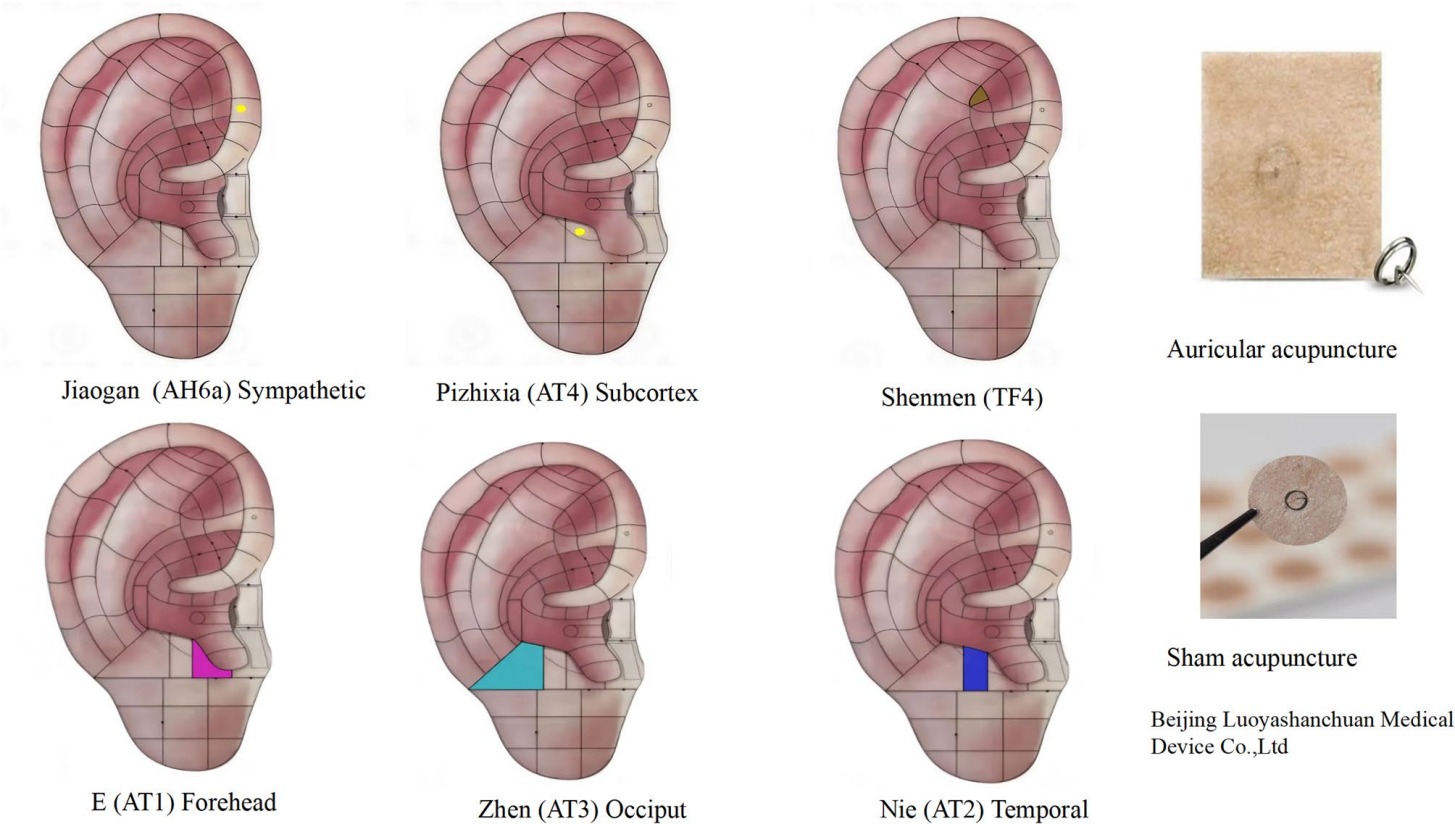
Acupuncture Point Location Diagram and Auricular Acupuncture Devices Used in the Study. The national standard auricular acupoint refers to the name and standard positioning of auricular acupoint (GB/T 13734 − 2008) issued by the National Standards Administration of the People’s Republic of China. Auricular acupuncture products from Beijing Luoyashanchuan Medical Device Co., Ltd. were used in this study

The acupuncture prescription included the following acupoints: Jiaogan (AH6a, Sympathetic); Pizhixia (AT4, Subcortex); Shenmen (TF4); E (AT1, Forehead); Zhen (AT3, Occiput); Nie (AT2, Temporal). Acupoint selection was strictly guided by headache lateralization, in accordance with our study protocol: Ipsilateral acupoints were needled for unilateral headache presentation, while bilateral acupoints were employed in cases of bilateral headache distribution. In this study, auricular acupuncture products from Beijing Luoyashanchuan Medical Device Co., Ltd. were used. The auricular acupoints used in the study are shown in Fig. 2. The participants received acupuncture twice a week for 4 weeks (1 week as a course of treatment).

#### Auricular acupuncture (for the experimental group)

The treatment group was treated with auricular acupuncture. The specific operation steps were as follows: (1) Insertion of Auricular Acupuncture Needle: First, the participant was seated. The physician disinfected their fingers and the participant’s skin with iodophor. Then, the physician picked up the auricular acupuncture needle with forceps, accurately targeted the pre-selected acupoint, and gently inserted the needle into the skin. (2) Retention of Auricular Acupuncture Needle: After the needle was inserted, participants were instructed to keep the buried needle in the acupoint. They were advised to gently press the acupoint with their fingers, adjusting the pressure based on their comfort level. (3) Removal of Auricular Acupuncture Needle: The physician used forceps to slowly extract the needle from the skin and promptly placed the needle in a sharps container. Subsequently, the physician disinfected the treatment area to prevent potential infections.

#### Sham acupuncture (for the control group)

The sham acupuncture group used the same acupoints and operation procedures as the auricular acupuncture group. However, the sham acupuncture was performed using a 1-cm-diameter circular tape that lacked a sharp needle and was identical to the auricular acupuncture needle’s tape in appearance, color, and shape. The sham acupuncture devices used in the study are shown in Fig. 2.

#### Adverse events management

The recorder meticulously documented all adverse reactions and subsequently completed the case report form. Participants who withdrew from the study due to adverse events were included in the adverse event analysis. Specifically, their data were not only included in statistical evaluations but also meticulously recorded in the adverse event report form.

### Outcomes

#### Primary outcomes

The primary outcomes were the Visual Analogue Scale (VAS) score [13] and the headache index [14]. The VAS score was used in this study to assess headache severity. Participants marked their pain intensity on a 10-cm scale, with ‘no pain’ at one end and ‘worst possible pain’ at the other. These data were meticulously recorded in a headache diary. The headache index was calculated as pain intensity × frequency × duration. The VAS score was assessed at baseline, 24 h after the first treatment, the 2nd week, the 4th week, and the 8th week. The headache index was assessed at baseline, the 4th week, and the 8th week.

#### Secondary outcomes

Secondary outcomes included the Hamilton Anxiety Scale (HAMA), Hamilton Depression Scale (HAMD) scores [15], the blood flow velocity in the Anterior Cerebral Artery (ACA; V_ACA_), Middle Cerebral Artery (MCA; V_MCA_), and Posterior Cerebral Artery (PCA; V_PCA_); and the use of acute headache medication. The HAMA and HAMD scores were evaluated at the 4th week, 8th week, and 24th week after enrolment. The use of acute headache medication was recorded at baseline and the 4th week after enrolment.

Transcranial Doppler ultrasound (Delikai Medical Equipment Co., Ltd., Model: EMS-9UA) was used to examine the major intracranial arteries of the participants, and the blood flow velocity in the ACA (V_ACA_), MCA (V_MCA_), and PCA (V_PCA_) was measured [16, 17]. The probe frequency was set to 2.0 MHz, and blood flow velocity data were assessed at baseline and the 4th week.

### Evaluation of efficacy

The therapeutic effect percentage was calculated using the headache index, in accordance with the criteria outlined in the “Head Wind Syndrome: Diagnostic and Efficacy Evaluation Criteria” [18]. The efficacy index was calculated according to the following formula: Efficacy index = (pre-treatment score - post-treatment score)/pre-treatment score × 100%. The efficacy levels were defined as follows: Basic Recovery: The efficacy index is ≥ 90% and < 100%; Marked Efficacy: The efficacy index is ≥ 55% and < 90%; Effective: The efficacy index is ≥ 20% and < 55%; Ineffective: The efficacy index is < 20%. The total effective rate (%) was calculated as the sum of the basic recovery rate, the rate of marked efficacy, and the effective rate.

## Results

### Participants

A total of 80 participants who met the inclusion and exclusion criteria were recruited for this study, with 40 participants in each group. Four participants (10% of the initial treatment group) and three participants (7.5% of the initial control group) withdrew from the study due to fear of acupuncture. The remaining 73 participants completed the treatment.

The mean age of the participants was (44.90 ± 11.33) years. The mean duration of headache was (11.05 ± 3.35) days per month. There were 32 males (43.84%) and 41 females (56.16%). Among them, 27 participants (36.99%) had menstruation-related headache, and these participants accounted for 65.85% of the female participants. All 73 participants used acute headache medication. The baseline characteristics of the two groups were balanced (*P* > 0.05). The figures are summarized in Table 1.

**Table 1:** Baseline characteristics of trial participants

	Total (*n* = 73)	Auricular acupuncture (*n* = 36)	Sham acupuncture (*n* = 37)	*P* value
Age (mean ± SD, years)	44.90 ± 11.33	45.81 ± 11.87	44.03 ± 10.86	0.506^a^
Duration (mean ± SD, days per month)	11.05 ± 3.35	10.92 ± 3.52	11.18 ± 3.22	0.744^a^
Gender				
Male n (%)	32 (43.84)	15 (41.66)	17 (45.95)	0.713^b^
Female n (%)	41 (56.16)	21 (58.33)	20 (54.05)	
Usage of acute headache medications, n (%)	67 (91.78)	32 (88.89)	35 (94.59)	0.430^c^
Menstrual-related migraine, n (%)	27 (36.99)	15 (41.67)	12 (32.43)	0.472^c^

*Abbreviations*: *SD *Standard deviation

^a^*p* values based on T test

^b^*p* values based on chi-squared test

^c^*p* values based on Fisher’s exact test

### Comparison of observation outcomes

#### VAS score

Before treatment, no significant differences in VAS scores were observed between the groups (*P* = 0.388; 4 (3.25, 5) vs. 4 (3, 4)). Significant differences in VAS scores were observed at 24 h after treatment (*P* < 0.001; 2 (1.25, 2) vs. 3 (2, 3.5)) and the 2nd week (*P* = 0.004; 2 (1.25, 2) vs. 2 (2, 3)) after treatment. At the 4th week, VAS scores between the groups remained significantly different (*P* = 0.015; 2 (1, 2) vs. 2 (1.5, 3)). However, at the 8th week after treatment, the VAS scores between the groups were no longer significantly different (*P* = 0.414; 2 (1, 2) vs. 2 (1, 2)).

**Fig. 3:**
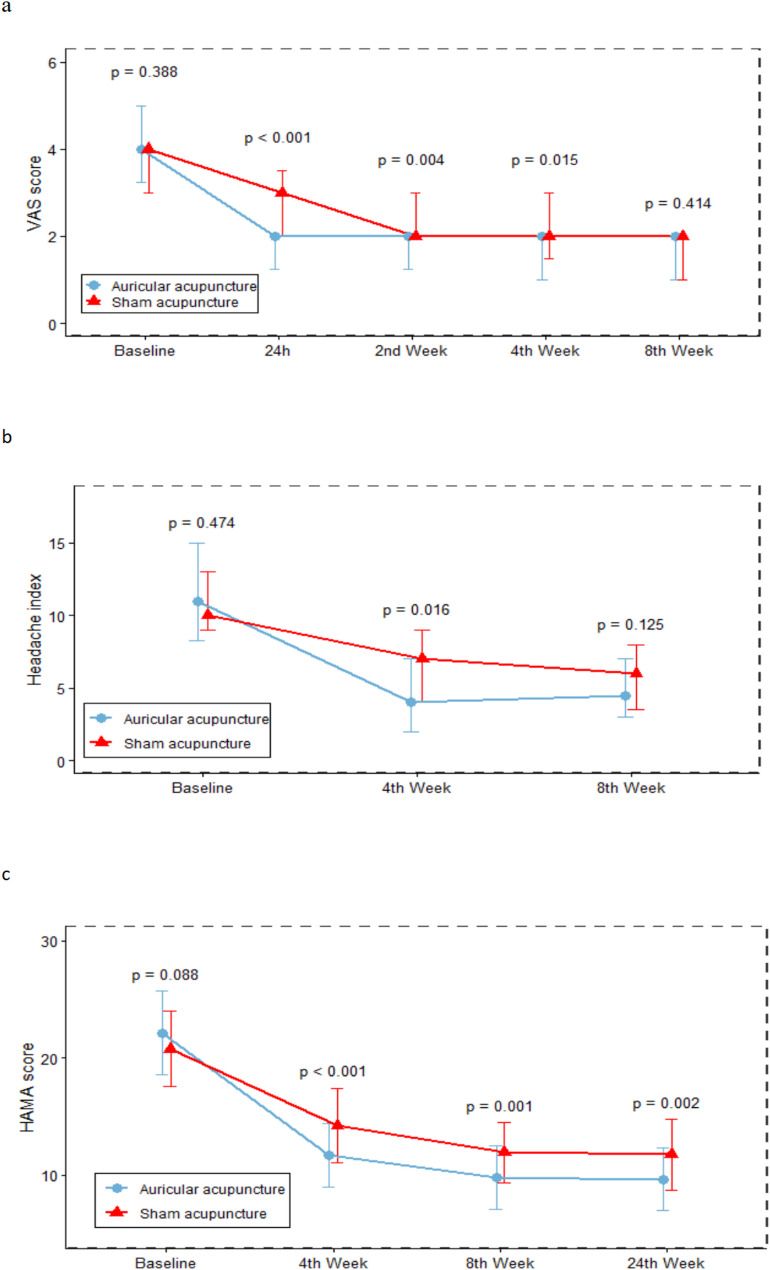
Time course of the change from baseline in co-primary outcomes. **a** Time course of the change from baseline in VAS score, presented as median (QL [lower quartile]∼QU [upper quartile]) at each time point. **b** Time course of the change from baseline in Headache index, presented as median (QL∼QU) at each time point. **c** Time course of the change from baseline in HAMA score, presented as mean ± SD at each time point

Additionally, analysis of the change from baseline in VAS scores revealed significant differences between the groups at 24 h after treatment (*P* < 0.001; −2 (-2, -1.25) vs. -1 (-2, 0)) and the 8th week (*P* < 0.001; 0 (0, 0) vs. 0 (-1, 0)). The figures are summarized in Table 2; Fig. 3.

**Table 2:** Comparison of observation outcomes

Outcomes	Group		*P* value
	Auricular acupuncture (*n* = 36)	Sham acupuncture (*n* = 37)	
VAS score M (QL∼QU)			
Baseline	4 (3.25, 5)	4 (3, 4)	0.388^a^
24 h	2 (1.25, 2)	3 (2, 3.5)	<0.001^a^*
2 nd Week	2 (1.25, 2)	2 (2, 3)	0.004^a^*
4 th Week	2 (1, 2)	2 (1.5, 3)	0.015^a^*
8 th Week	2 (1, 2)	2 (1, 2)	0.414^a^
The change from baseline in VAS Score M (QL∼QU)			
24 h	−2 (-2, -1.25)	−1 (-2, 0)	<0.001^a^*
2 nd Week	0 (−1, 0)	0 (−1, 0)	0.265^ac^
4 th Week	0 (−1, 0)	0 (−1, 0)	0.634^ac^
8 th Week	0 (0, 0)	0 (−1, 0)	<0.001^a^*
Headache index M (QL∼QU)			
Baseline,	11 (8.25, 15)	10 (9, 13)	0.474^a^
4 th Week	4 (2, 7)	7 (4, 9)	0.016^a^*
8 th Week	4.5 (3, 7)	6 (3.5, 8)	0.125^a^
The change from baseline in Headache index M (QL∼QU)			
4 th Week	−7 (-9, -6)	−3 (-7, -2)	0.002^c^*
8 th Week	0.5 (0, 1)	0 (−1, 0)	0.002^c^*
HAMA Score (mean ± SD)			
Baseline,	22.14 ± 3.61	20.76 ± 3.21	0.088^b^
4 th Week	11.69 ± 2.70	14.24 ± 3.20	<0.001^b^*
8 th Week	9.83 ± 2.71	11.95 ± 2.59	0.001^b^*
24 th Week	9.67 ± 2.65	11.76 ± 3.00	0.002^b^*
The change from baseline in HAMA Score (mean ± SD)			
4 th Week	−10.44 ± 4.59	−6.51 ± 2.92	<0.001^b^*
8 th Week	−1.86 ± 2.45	−2.30 ± 2.73	0.475^b^
24 th Week	−0.17 ± 0.65	−0.19 ± 2.04	0.950^b^
HAMD Score (mean ± SD)			
Baseline,	23.53 ± 3.93	22.59 ± 3.55	0.291^b^
4 th Week	14.25 ± 2.68	15.89 ± 3.48	0.027^b^*
8 th Week	14.14 ± 2.84	15.54 ± 3.23	0.053^b^
24 th Week	14.17 ± 2.94	15.41 ± 3.08	0.083^b^
The change from baseline in HAMD Score (mean ± SD)			
4 th Week	−9.28 ± 3.01	−6.70 ± 2.41	<0.001^b^*
8 th Week	−0.11 ± 0.52	−0.35 ± 1.14	0.252^b^
24 th Week	0.03 ± 0.51	−0.14 ± 0.98	0.376^b^
V_ACA_ M (QL∼QU)			
Baseline,	81.5 (80.25, 82.75)	81 (80, 82.5)	0.878^ac^
4 th Week	85.5 (85, 86)	83 (83, 84)	<0.001^a^*
The change from baseline in V_ACA_ M (QL∼QU)			
4 th Week	4 (3, 5)	3 (1, 3)	<0.001^a^*
V_MCA_ M (QL∼QU)			
Baseline,	107 (106, 108)	107 (106, 108)	0.942^c^
4 th Weeks	113 (111, 115)	113 (110.5, 115)	0.519^c^
The change from baseline in V _MCA_			
4 th Week	6 (4, 9)	6 (4, 9)	0.641^c^
V_PCA_ M (QL∼QU)			
Baseline,	74 (73, 75)	74 (73, 75)	0.937^c^
4 th Week	82 (81, 83)	78 (77, 79)	<0.001^c^*
The change from baseline in V_PCA_ M (QL∼QU)			
4 th Week	8 (8, 8)	4 (4, 4)	<0.001^a^*
Usage of acute headache medications M (QL∼QU)			
Baseline,	14.5 (12, 17)	14 (11, 16.5)	0.485^c^
4 th Week	8 (7, 10)	9 (8, 11)	0.030^c^*
The change from baseline in Usage of acute headache medications M (QL∼QU)			
4 th Week	−5 (-8, -4)	−4 (-8, -2)	0.069^c^

*Abbreviations*: *SD* Standard deviation, *M* Median, the middle value of a dataset when ordered from smallest to largest, *QL* represents the lower quartile (Q1), the 25th percentile of the data, QU represents the upper quartile (Q3), the 75th percentile of the data

* *p* values less than 0.05

^a^*p* values based on Kruskal Wallis test

^b^*p* values based on T test

^c^*p* values based on Mann-Whitney U test

#### Headache index

Before treatment, no significant differences in headache index were observed between the groups (*P* = 0.474; 11 (8.25, 15) vs. 10 (9, 13)). At the 4th week, significant differences in headache index were observed between the groups (*P* = 0.016; 4 (2, 7) vs. 7 (4, 9)). However, at the 8th week, the headache index between the groups was no longer significantly different (*P* = 0.125; 4.5 (3, 7) vs. 6 (3.5, 8)).

Analysis of the change from baseline in headache index revealed significant differences between the groups at the 4th week and the 8th week (both *P* = 0.002; −7 (-9, -6) vs. -3 (-7, -2); 0.5 (0, 1) vs. 0 (-1, 0)). The figures are summarized in Table 2; Fig. 3.

#### HAMA score

Before treatment, no significant differences in HAMA score were observed between the groups (*P* = 0.088; 22.14 ± 3.61 vs. 20.76 ± 3.21). At the 4th week, the 8th week, and the 24th week, the HAMA score between the groups was significantly different. At the 4th week (*P* < 0.001; 11.69 ± 2.70 vs. 14.24 ± 3.20), the 8th week (*P* = 0.001; 9.83 ± 2.71 vs. 11.95 ± 2.59), and the 24th week (*P* = 0.002; 9.67 ± 2.65 vs. 11.76 ± 3.00).

Analysis of the change from baseline in HAMA score revealed significant differences between the groups at the 4th week (*P* < 0.001; −10.44 ± 4.59 vs. -6.51 ± 2.92). However, at the 8th and the 24th week, the change from baseline in HAMA score between the groups was no longer significantly different (*P* = 0.475; *P* = 0.950, respectively). The figures are summarized in Table 2.

#### HAMD score

Before treatment, no significant differences in HAMD score were observed between the groups (*P* = 0.291; 23.53 ± 3.93 vs. 22.59 ± 3.55). At the 4th week, the HAMD score between the groups was significantly different (*P* = 0.027; 14.25 ± 2.68 vs. 15.89 ± 3.48). However, at the 8th and the 24th week, the HAMD score between the groups was no longer significantly different (*P* = 0.053 at the 8th week; *P* = 0.083 at the 24th week).

Analysis of the change from baseline in HAMD score revealed significant differences between the groups at the 4th week (*P* < 0.001; −9.28 ± 3.01 vs. -6.70 ± 2.41). However, at the 8th and the 24th week, the change from baseline in HAMD score between the groups was no longer significantly different (*P* = 0.252 at the 8th week; *P* = 0.376 at the 24th week). The figures are summarized in Table 2; Fig. 3.

#### V_ACA_, V_MCA_ and V_PCA_

Before treatment, no significant differences in V_ACA_ were observed between the groups (*P* = 0.878; 81.5 (80.25, 82.75) vs. 81 (80, 82.5)). However, at the 4th week, significant differences in V_ACA_ were observed between the groups (*P* < 0.001; 85.5 (85, 86) vs. 83 (83, 84)). Analysis of the change from baseline in V_ACA_ revealed significant differences between the groups at the 4th week (*P* < 0.001; 4 (3, 5) vs. 3 (1, 3)).

Before treatment, no significant differences in V_MCA_ were observed between the groups (*P* = 0.942; 107 (106, 108) vs. 107 (106, 108)). At the 4th week, the V_MCA_ between the groups was not significantly different (*P* = 0.519; 113 (111, 115) vs. 113 (110.5, 115)). Analysis of the change from baseline in VMCA revealed no significant differences between the groups at the 4th week (*P* = 0.641).

Before treatment, no significant differences in V_PCA_ were observed between the groups (*P* = 0.937; 74 (73, 75) vs. 74 (73, 75)). However, at the 4th week, significant differences in V_PCA_ were observed between the groups (*P* < 0.001; 82 (81, 83) vs. 78 (77, 79)). Analysis of the change from baseline in V_PCA_ revealed significant differences between the groups at the 4th week (*P* < 0.001; 8 (8, 8) vs. 4 (4, 4)). The results are summarized in Table 2.

### Usage of acute headache medications

Before treatment, no significant differences in the usage of acute headache medications were observed between the groups (*P* = 0.485; 14.5 (12, 17) vs. 14 (11, 16.5)). At the 4th week, significant differences in the usage of acute headache medications were observed between the groups (*P* = 0.030; 8 (7, 10) vs. 9 (8, 11)). However, analysis of the change from baseline in the usage of acute headache medications revealed no significant differences between the groups at the 4th week (*P* = 0.069). The figures are summarized in Table 2.

### Clinical efficacy

The treatment period was 4 weeks. The effective rate was 91.67% in the auricular acupuncture group and 64.86% in the sham-acupuncture group. There was a statistically significant difference in the total effective rate between the two groups (*P* = 0.009).

### Evaluation of safety

During clinical treatment, one adverse event occurred in both groups, which was mild and resolved with rest. There was no significant difference in the incidence of adverse reactions between the two groups (*P* = 1.000), indicating that the treatment regimen was safe and reliable.

### Treatment credibility

A range of guesses about the type of treatment reflected the participants’ perceptions at each follow-up assessment (Table 3). The questionnaire contained five factors: guesses of treatment type at the 4th, 8th, and 24th weeks, treatment logic, and recommendation to others. The KMO value was 0.892, and Bartlett’s sphericity test was significant (*P* < 0.001), affirming the viability of the questionnaire.

**Table 3:** Serial guess of treatment type and treatment credibility at the end of the study

Outcomes	Group		*P* value
	Auricular acupuncture (*n* = 36)	Sham acupuncture (*n* = 37)	
Guess of treatment type at 4 th week, n (%)			
Auricular acupuncture	7 (19.44)	5 (13.51)	0.780^a^
Sham acupuncture	5 (13.89)	5 (13.51)	
Don’t know	24 (66.67)	27 (72.97)	
Guess of treatment type at 8 th week, n (%)			
Auricular acupuncture	8 (22.22)	5 (13.51)	0.498^a^
Sham acupuncture	5 (13.89)	8 (21.62)	
Don’t know	23 (63.89)	24 (64.86)	
Guess of treatment type at 24 th week, n (%)			
Auricular acupuncture	17 (47.22)	7 (18.92)	0.036^a^*
Sham acupuncture	4 (11.11)	7 (18.92)	
Don’t know	15 (41.67)	23 (62.16)	
Treatment credibility at the end of the study, n (%)			
Treatment logical	32 (88.89)	30 (81.08)	0.004^a^*
Recommendation to others	32 (88.89)	29 (78.38)	0.345^b^

*p values less than 0.05

^a^*p* values based on chi-squared test

^b^*p* values based on Fisher’s exact test

At week 4, the BBI was approximately 0.165, indicating that the proportion of participants who correctly guessed their treatment allocation was relatively low. The JBI was approximately 0.944 (*P* = 0.780), suggesting little difference in guessing between the two groups and that the trial’s blinding was relatively successful.

At week 8, the BBI was 0.178, indicating the blinding effect remained acceptable but had slightly declined compared to week 4. The JBI was 0.917 (*P* = 0.498), consistent with minimal group differences in guessing and an acceptable level of blinding.

At week 24, the BBI was approximately 0.329, indicating that about 32.9% of the participants correctly guessed their type of treatment. The JBI was approximately 0.722 (*P* = 0.036), indicating an increased difference in guessing between the two groups compared to earlier assessments and that the trial’s blinding was compromised to some extent.

Collectively, these findings suggest that blinding was most effective in the early stage (week 4), remained acceptable in the middle stage (week 8), but was compromised in the late stage (week 24). There was no significant difference in recommendation to others between the two groups (*P* = 0.345).

## Discussion

Headache is a multifactorial symptom with heterogeneous pathophysiology and represents a major clinical and public health challenge. Globally, tension headache (TTH) is the most prevalent primary headache disorder, accounting for 42% of all headache cases. Although most headaches are self-limiting, TTH is characterized by recurrent episodes of mild to moderate bilateral pain, often described as a “pressure-like” sensation. The symptoms of chronic TTH persist and severely impact patients’ quality of life [19].

TTH is the most common subtype of primary headache and a prevalent neurological disorder in the general population. The specific classification of TTH includes infrequent episodic TTH (IETTH), frequent episodic TTH (FETTH), and chronic TTH. Among these subtypes, IETTH generally has a minimal impact on patients’ daily activities [5, 6]. This subtype rarely requires pharmacologic treatment, as symptoms can typically be managed through lifestyle modifications (e.g., stress reduction and improved sleep hygiene) and non-pharmacological interventions. Conversely, frequent episodic TTH (FETTH) is associated with significant functional disability. FETTH is characterized by severe pain during acute attacks, which can disrupt work productivity and daily functioning. Given the substantial impact on quality of life, timely pharmacologic intervention is essential to alleviate symptoms and improve patient outcomes, in accordance with current evidence-based guidelines. Due to the neglect of early intervention for IETTH and FETTH, the progression of these conditions to chronic TTH is quite common. Therefore, timely acupuncture treatment for FETTH is of paramount importance. It is crucial not only for alleviating pain symptoms and the associated psychological and mental disorders induced by these symptoms but also for preventing the development of chronic TTH [5, 12].

Current clinical guidelines recommend NSAIDs as first-line therapy for acute TTH, while tricyclic antidepressants are preferred for chronic TTH management. However, pharmacological interventions face limitations, such as gastrointestinal complications, tolerance development, and the risk of medication-overuse headache, which necessitate alternative therapeutic strategies [20].

Therefore, it is crucial to explore alternative therapies for the effective treatment of TTH. Currently, commonly used alternative therapies include electromyography biofeedback therapy [21], cognitive behavioral therapy [22], cutaneous massage, and other approaches [23]. However, these therapeutic approaches have certain limitations. For example, they are time-consuming and require high patient compliance. Given these limitations, we have considered acupuncture therapy, a traditional medical approach. Acupuncture therapy for TTH has shown significant clinical efficacy [24]. In clinical practice, it has been widely recognized that acupuncture treatment is less effective for chronic TTH than for IETTH and FETTH. This reduced efficacy may be due to the more complex pain mechanisms underlying chronic TTH, involving greater central sensitization and neuroplastic changes. Given these challenges, we emphasize the importance of early clinical intervention to prevent the progression of IETTH and FETTH to chronic TTH. Early identification and treatment can help maximize the benefits of acupuncture, improve therapeutic outcomes, and enhance patients’ quality of life.

According to the theory of meridians and collaterals in TCM, the ear serves as a convergence point for multiple meridians. Modern research has confirmed that the vagus nerve projects directly to the nucleus tractus solitarius (NTS). Stimulation of the ear can activate the vagus nerve-NTS pathway. Auricular acupuncture, auricular pressure, and transcutaneous electrical auricular stimulation are all effective in modulating pain [25]. Auricular acupoints are reaction points located on the auricle. In disease states, they may show pathological changes like tenderness or form nodules. TCM considers these auricular points as therapeutic sites for stimulation.

Vosviewer analysis identified the top 10 auricular acupoints for headache treatment as Shenmen, Subcortex, Temporal, Sympathetic, Liver, Occipital, Pancreas, and Gallbladder, Endocrine, Frontal, and Kidney [26]. The auricular branch of the vagus nerve innervates the external auditory canal, inner ear, and auricular skin, serving as the only somatic afferent branch of the vagus nerve. Moreover, the auricular area is exclusively innervated by its auricular branch [27]. Of the top 10 auricular acupoints for headache treatment, 7 are located in the distribution area of the auricular branch of the vagus nerve. The total frequency of application for these points is higher than that for points not innervated by the vagus nerve.

Auricular therapy demonstrates efficacy in headache management, although its mechanisms of action remain incompletely elucidated. Current evidence suggests that its analgesic effects may arise from stimulation of the auricular branch of the vagus nerve. Multiple studies propose vagus nerve modulation as a plausible pathway for headache alleviation through auricular interventions. A meta-analysis of 17 RCTs identified 20 commonly used analgesic auricular points, 15 of which are located in regions innervated by the auricular branch of the vagus nerve or exhibit dual innervation with other neural pathways [28]. The theoretical framework established by Nogier et al. supports the therapeutic impact of the auricular liver zone on migraine pathophysiology [29]. Subsequent acupuncture contact testing, which involved percutaneous stimulation of scalp and auricular dermatomes, determined optimal sites for migraine pain relief. Results demonstrated that maximal analgesic efficacy occurred at tender points within the ipsilateral upper auricular concha (Liver point) and contralateral anteromedial tragus (Subcortex point). Semi-permanent needle implantation at these loci achieved sustained control of migraine symptoms [30].

The inner ear is densely innervated by a rich network of sensory nerves, primarily the auricular branch of the vagus nerve, the auricular nerve, the occipital nerve, and the auriculotemporal nerve. These nerves converge and overlap within the ear, creating a complex network [31]. Auricular therapy effectively stimulates neural activity and modulates sensory perception in the innervated regions [32]. The vagus nerve comprises both somatic and visceral afferent and efferent fibers. The auricular branch of the vagus nerve, which is the only somatic afferent branch, primarily innervates the external auditory canal, inner ear, and surrounding auricular skin [33]. The vagus nerve is closely connected to the central nervous system, which contains a descending pain modulation system.Neurons in the periaqueductal gray (PAG) contain endorphins and opioid receptors.Auricular stimulation can activate the vagus nerve, indirectly stimulating the PAG to increase pain threshold and produce analgesia [34]. The dorsal raphe nucleus (DR) is a key nuclear group responsible for the central modulation of pain. Serotonin (5-HT), a neurotransmitter involved in pain modulation, is synthesized and released in the DR [35]. Vagus nerve stimulation alters the firing of serotonergic neurons in the descending pathways from the dorsal raphe nucleus (DR), causing the release of serotonin (5-HT). This increases central 5-HT levels, enhances the blood-brain barrier’s permeability to 5-HT, and relieves headaches. The locus coeruleus (LC), which serves as a relay nucleus for the spinal cord, thalamus, and hypothalamus, synthesizes and releases norepinephrine (NE), another key neurotransmitter for pain modulation [36]. Auricular therapy may modulate pain by releasing Calcitonin gene-related peptide (CGRP) and other substances involved in neuroinflammation. CGRP is implicated in pain signaling and inflammation, acting on blood vessels and immune cells to influence pain perception and inflammatory processes. Auricular therapy could alleviate pain by modulating CGRP release and activity [37].

Auricular acupuncture, also known as intradermal acupuncture, was used in this study. Specially designed small needles, which were fixed to the auricular points with medical tape, provided long-lasting local stimulation. Compared with other therapies, auricular acupuncture had several advantages, including prolonged stimulation duration, high stimulation intensity, unrestricted patient movement, and avoiding needle-related anxiety. Therefore, auricular acupuncture was considered an effective method for treating TTH. The acupuncture prescription included the following points: Jiaogan (AH6a, Sympathetic), located at the junction of the anterior lower segment of the contralateral helix and the inner edge of the second helix, can regulate autonomic nerve function and relieve headache symptoms. Pizhixia (AT4, Subcortex), located on the medial side of the contralateral auricle screen, can regulate the function of the cerebral cortex and reduce cortical tension, which is key to relieving pain. Shenmen (TF4), located in the upper 1/3 of the triangular fossa, can relieve anxiety and depression. E (AT1, Forehead), located in the anterior region of the contralateral auricle screen, has sedative and analgesic effects and corresponds to the treatment of frontal headaches. Nie (AT2, Temporal), located in the middle region of the contralateral auricle screen, has sedative and analgesic effects and corresponds to the treatment of temporal headaches. Zhen (AT3, Occiput), located in the posterior region of the contralateral auricle screen, has sedative and analgesic effects and corresponds to the treatment of occipital headaches.

This study found that the sham acupuncture group exhibited some therapeutic effects, which are believed to be related to the stimulation of the patient’s acupoints. Sham acupuncture may alleviate headache symptoms by stimulating peripheral nerve endings and regulating nerve function. The mechanical stimulation of acupuncture is thought to activate specific brain regions closely related to pain regulation, such as the brainstem and thalamus. Through these neural regulatory mechanisms, sham acupuncture can reduce the frequency and intensity of headaches. Additionally, sham acupuncture may alleviate headaches by enhancing local microcirculation dynamics. These hemodynamic changes can help alleviate the inflammatory response associated with headaches. Moreover, sham acupuncture may trigger the release of endogenous opioid peptides, such as β-endorphin, which bind to central opioid receptors and inhibit the transmission of noxious signals in the neural circuits involved in pain processing [38].

Therefore, auricular acupuncture is a feasible and effective treatment option for FETTH. It can effectively reduce the frequency and severity of FETTH episodes, decrease the use of painkillers, and improve quality of life. Additionally, auricular acupuncture has shown promise in alleviating anxiety and depression related to FETTH and has demonstrated a favorable safety profile. Given these findings, auricular acupuncture represents a valuable non-pharmacological treatment option for FETTH.

This study has several limitations. Firstly, issues related to participant compliance have limited the sample size. Secondly, data collection was confined to a single hospital, which may lead to bias. Thirdly, the unique nature of acupuncture therapy poses challenges for implementing a double-blind design, potentially influencing treatment outcomes subjectively. Fourthly, time constraints of the study hindered long-term observation of its efficacy and the collection of biochemical markers. Finally, the mechanism underlying auricular acupuncture for treating FETTH remains unclear. Therefore, it is recommended that future multi-center, large-sample, and long-term follow-up randomized controlled trials (RCTs) be conducted to explore its efficacy mechanisms and provide more valuable insights for future treatments.

## Conclusion

In summary, this single-blind, randomized controlled clinical trial provides evidence for the efficacy and safety of auricular acupuncture in treating FETTH among the Xianyang population. It also offers a reference for clinical practitioners considering auricular acupuncture for treating FETTH. However, further research is needed to elucidate the mechanisms underlying auricular acupuncture in treating FETTH.

## Data Availability

All deidentified individual participant data collected during this trial will be made publicly accessible upon publication and will remain available indefinitely. Researchers may request access to these datasets by submitting a formal proposal to the principal investigator, MD. Ziwen Xu, via email at 707869505@qq.com. Approved requestors will be required to execute a data access agreement prior to obtaining the anonymized data.

## Abbreviations

TTH: Tension type headache
FETTH: Frequent episodic tension type headache
IETTH: Infrequent episodic Tension type headache
HAMA: Hamilton Anxiety Scale
HAMD: Hamilton Depression Scale
VAS: Vsual analogue scale
RCT: Randomized controlled trial
STRICTA: Standards for Reporting Interventions in Clinical Trials of Acupuncture
CONSORT: Consolidated Standards of Reporting Trials
ICHD: International Classification of Headache Disorders
SD: Standard Deviation
M: Median
QL: Quartile Lower
QU: Quartile Upper
KMO: Kaiser-Meyer– Olkin
JBI: James Blinding Index
BBI: Bang Blinding Index
ITT: Intention-to-treat
ACA: Arteria cerebri rostralis
MCA: Middle cerebri rostralis
PCA: Posterior cerebri rostralis
V_ACA_: Blood flow velocity in the anterior cerebral artery
V_MCA_: Blood flow velocity in the middle cerebral artery
V_PCA_: Blood flow velocity in the posterior cerebral artery
NTS: Nucleus tractus solitarii
LC: Locus Coeruleus
5-HT: 5-hydroxytryptamine
DR: Dorsal Raphe
NE: Norepinephrine
PAG: Periaqueductal gray
CGRP: Calcitonin gene-related peptide
